# Severe Hypoglycemia: Is It Still a Threat for Children and Adolescents With Type 1 Diabetes?

**DOI:** 10.3389/fendo.2020.00609

**Published:** 2020-09-15

**Authors:** Tatsuhiko Urakami

**Affiliations:** Department of Pediatrics, Nihon University School of Medicine, Tokyo, Japan

**Keywords:** severe hypoglycemia, children and adolescents, type 1 diabetes, risk factor, advanced technology

## Abstract

Severe hypoglycemia is defined as a condition with serious cognitive dysfunction, such as a convulsion and coma, requiring external help from other persons. This condition is still lethal and is reported to be the cause of death in 4–10% in children and adolescents with type 1 diabetes. The incidence of severe hypoglycemia in the pediatric population was previously reported as high as more than 50–100 patient-years; however, there was a decline in the frequency of severe hypoglycemia during the past decades, and relationship with glycemic control became weaker than previously reported. A lot of studies have shown the neurological sequelae with severe hypoglycemia as cognitive dysfunction and abnormalities in brain structure. This serious condition also provides negative psychosocial outcomes and undesirable compensatory behaviors. Various possible factors, such as younger age, recurrent hypoglycemia, nocturnal hypoglycemia, and impaired awareness of hypoglycemia, are possible risk factors for developing severe hypoglycemia. A low HbA_1c_ level is not a predictable value for severe hypoglycemia. Prevention of severe hypoglycemia remains one of the most critical issues in the management of pediatric patients with type 1 diabetes. Advanced technologies, such as continuous glucose monitoring (CGM), intermittently scanned CGM, and sensor-augmented pump therapy with low-glucose suspend system, potentially minimize the occurrence of severe hypoglycemia without worsening overall glycemic control. Hybrid closed-loop system must be the most promising tool for achieving optimal glycemic control with preventing the occurrence of severe hypoglycemia in pediatric patients with type 1 diabetes.

## Introduction

Hypoglycemia is a commonly observed acute complication in the management of type 1 diabetes. It is a major barrier to achieve optimal glycemic control (1) and may affect quality of life in the patients (2). Minimizing hypoglycemia is an important objective in the management of type 1 diabetes, and this can be attained by evaluating the risk factors and preventing them, although intensive glycemic management (3).

Severe hypoglycemia is defined as a condition with serious cognitive dysfunction, such as a convulsion and coma, requiring external help from other persons to provide glucose and glucagon or take other correction assistance. Severe hypoglycemic coma is defined as the subgroup of severe hypoglycemia related to a convulsion or unconsciousness (4). Severe hypoglycemia is still lethal and is reported to be the cause of death in 4% to 10% (5–7). It may be associated with permanent brain damage and is related to cognitive dysfunction and abnormalities in brain structure particularly in young children with type 1 diabetes (8–15). The International Society for Pediatric and Adolescent Diabetes Clinical Practice Consensus Guidelines 2018 has recommended that a target of HbA_1c_ should be <7.0% (53 mmol/mol) in patients who can access contemporary technologies of insulin treatment and the potency to regular self-checking blood glucose and/or the use of continuous glucose monitoring (CGM) (16). Whereas, careful attention must be poured to avoid severe hypoglycemia, glucose targets must be increased in patients with the risk factors for severe hypoglycemia (4, 17).

The main purpose of this review is to evaluate possible risk factors and the neurological sequelae in severe hypoglycemia and to introduce the advanced technologies to minimize the occurrence of severe hypoglycemia in children and adolescents with type 1 diabetes.

## Research Methods

Briefly, we performed the literature research by MEDLINE and EMBASE covering the period between 1980 and 2019. The search terms were “type 1 diabetes,” “children,” “adolescents,” and “hypoglycemia” or “severe hypoglycemia.” Language restriction was applied in English.

## Incidence

High incidence of severe hypoglycemia was shown by the Diabetes Control and Complications Trial (DCCT) in 1997 (18); i.e., the incidence of hypoglycemia that needed treatment assistance was 61.2 per 100 patient-years in patients receiving intensive treatment and 18.7 per 100 patient-years in those receiving conventional treatment, respectively, with a relative risk of 3.28. The relative risk for coma and/or seizure was 3.02 for intensive treatment. High incidence was also demonstrated in large pediatric cohorts in Australia (19) and Colorado (20) in the early 2000s. However, the incidence has been decreasing over time. A population-based cohort of Western Australia demonstrated that the incidence of severe hypoglycemia was 17.3 per 100 patient-years in 2001 and 5.8 per 100 patient-years in 2006, and a 12% annual rate of decrease was observed during the study period (21). A similar decreased trend was also observed in children and adolescents in Germany and Australia (22) and in Japan (23). A recent Italian study conducted in 29 diabetes centers during 2011–2012 reported less incidence of 7.7 per 100 patient-years (24), whereas another Italian-center study showed higher incidence of 12.6 per 100 patient-years in 1990 and 16.5 per 100 patient-years in 2010, respectively (Table 1). Development of treatment regimens might contribute to decrease in the incidence of severe hypoglycemia; however, despite the advent of new insulin regimens, severe hypoglycemia still remained a relevant risk and a current threat for patients with type 1 diabetes and their family members (25).

**Table 1:** Incidence of severe hypoglycemia over time.

**Report**	**Year**	Incidence^*^	**References**
DCCT	1984–1993		(18)
Conventional		18.7	
Intensive		61.2	
Bulsara MK	1992	7.8	(19)
	2002	16.6	
Rewers A	1996–2000	19.0	(20)
O'Connell SM	2001	17.3	(21)
	2006	5.8	
Karges B	1995	20.7	(22)
	2012	3.6	
Urakami T	2003–2013	4.0	(23)
Cherubini V	2011–2012	7.7	(24)
Maltoni G	1990	12.6	(25)
	2010	16.5	

* *Per 100 patient-years*.

On the other hand, previous studies showed that high incidence of severe hypoglycemia was related to a lower HbA_1c_ level (18, 26); however, this association has recently weakened as reported in large longitudinal cohorts (21, 27, 28). A cross-sectional analysis of 3 contemporary pediatric diabetes registry databases showed no inverse correlation between a mean HbA_1c_ level and risk factors for severe hypoglycemia in children and adolescents with type 1 diabetes (29). It is possible that the advanced technologies over the past decades could be enabling better glycemic control without increase in the risk of severe hypoglycemia. Such advances could include the introduction and increased use of insulin analogs, insulin pump therapy, increased frequency of self-monitoring of blood glucose, and use of CGM.

## Morbidity

### Neurological Outcomes

#### Cognitive Function

Resent meta-analyses of the literature indicated that young patients with type 1 diabetes tended to show mildly lower overall intellectual function than healthy controls and that the domains of executive functions, learning, memory, and processing speed were also impaired (30, 31). On the other hand, larger difference in cognitive function was found in the subset of young patients with certain risk factors, including younger onset age and greater exposures to both severe hypoglycemia and hyperglycemia (32).

Several studies in pediatric patients with type 1 diabetes demonstrated frequent episodes of severe hypoglycemia were related to worse performance than healthy controls on certain attention tasks, such as overall cognitive function, and verbal and visual memory. Particularly, children with certain risk factors, including younger onset age and frequent episodes of severe hypoglycemia, tended to develop cognitive dysfunction. Lin et al. (10) found that severe hypoglycemia with early onset of type 1 diabetes below 6 years of age adversely affected verbal abilities, working memory, and processing speed later in life than healthy controls. Another study also showed that younger onset age (<5 years) was related to deficit of cognitive function (8). On the other hand, other studies demonstrated that frequent episodes of severe hypoglycemia particularly affected distinct memory function, when these episodes appeared before 5 years of age, and were related to full-scale IQ scores, processing speed, working memory, and perceptual reasoning (11, 12). Furthermore, severe hypoglycemia with a convulsion was associated with greater performance deficits, including attention tasks, overall cognitive function, and verbal and visual memory (33). Blasetti et al. (34) indicated that prior episodes of severe and frequent hypoglycemia were mostly related to decreased learning and memory in young patients with type 1 diabetes using a meta-analysis.

On the other hand, some studies have demonstrated that severe hypoglycemia is unlikely to affect cognitive function. The Epidemiology of Diabetes Interventions and Complications follow-up study, conducted 18 years after the DCCT, reported that cognitive function did not decrease over the extended period in the youngest patients, although relatively high frequencies of severe hypoglycemia (35). A cross-sectional (36) and longitudinal follow-up research in the same population-based cohort (37) also did not elucidate a decrease in full-scale IQ scores, although executive function and fluid intelligence may be insufficient. On the other hand, other studies demonstrated that cognitive dysfunction also occurred with hyperglycemia other than hypoglycemia (9, 38–40). A large study of younger children (4–10 years of age) with a short period of type 1 diabetes (mean 2.5 years) reported cognitive differences than age-matched healthy children (40). There were differences in full-scale IQ scores and executive functioning even after adjustment of parent IQ scores and internalizing mood symptom levels. The degree of exposure to hyperglycemia was relatively related to performance in these domains. The long-duration impact of hyperglycemia may play an additional role for cognitive outcomes in pediatric patients with type 1 diabetes.

#### Brain Structure

**Figure 1:**
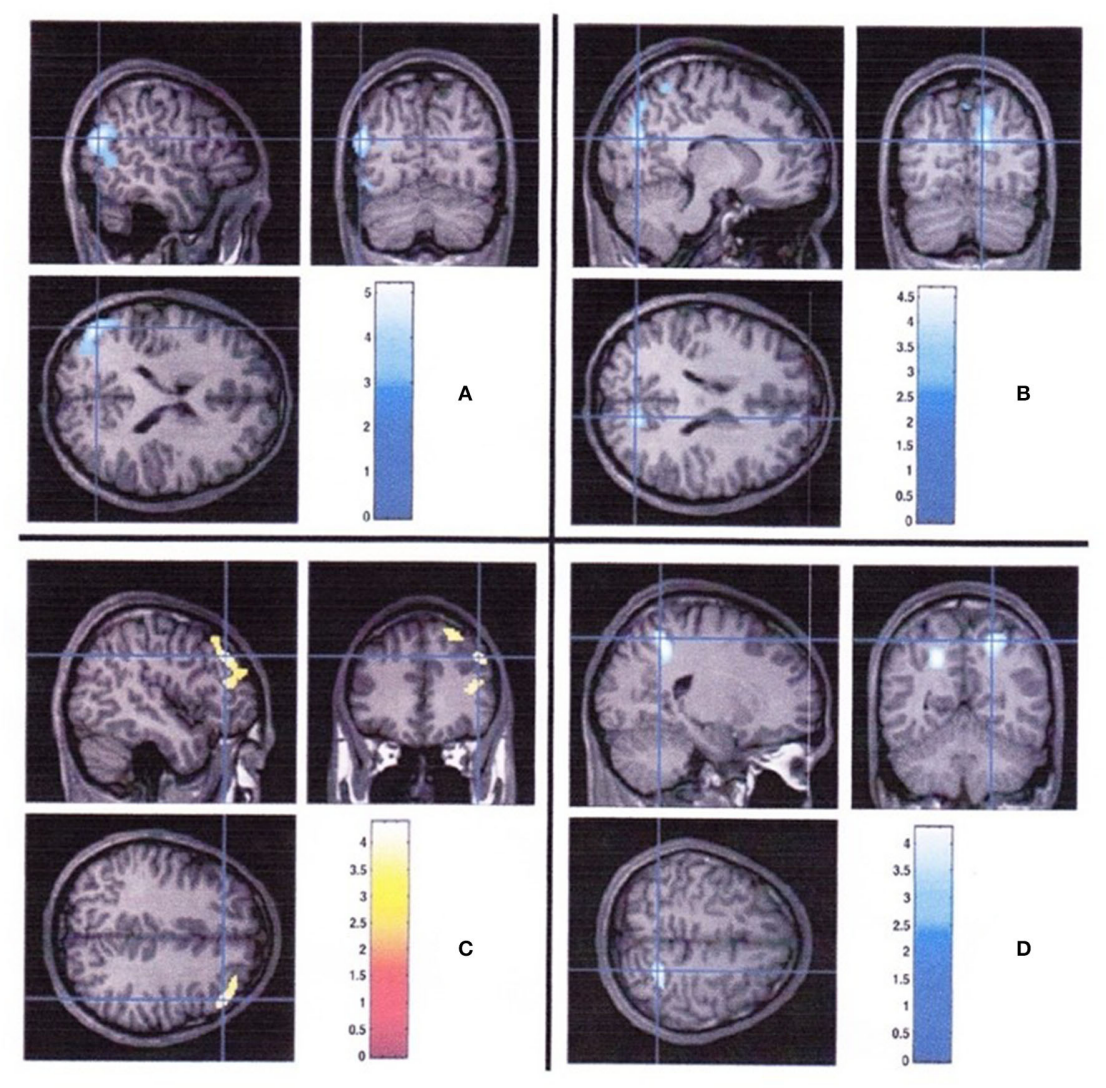
Regional brain volume differences associated with hyperglycemia and severe hypoglycemia in youth with type 1 diabetes (15). **(A)** Regions with smaller gray matter volume in diabetic youth with severe hypoglycemia compared with those in diabetic youth without severe hypoglycemia. **(B)** Regions of less gray matter, **(C)** more gray matter, and **(D)** less white matter associated with greater hyperglycemia exposure.

The association of structural abnormalities of brain accompanied by severe hypoglycemia has been shown, although there is increasing evidence that the brain changes were observed even without significant episodes of hypoglycemia among young patients with type 1 diabetes. Pell et al. (41) reported an interaction between age and brain volume with youth with type 1 diabetes but with the occurrence of dysglycemia. Greater hippocampal volumes (14) and decreased gray and white matter volumes were observed in children experienced hypoglycemic seizures (12). However, another study showed that brain changes were observed both with hypoglycemia and with hyperglycemia. Episodes of severe hypoglycemia were related to decreased gray matter volume in the left superior temporal region, whereas frequent episodes of hyperglycemia were related to decreased gray matter volume in the right cuneus and precuneus, decreased white matter volume in the right posterior parietal region, and increased gray matter volume in the right prefrontal region (15) (Figure 1). These findings suggest that regional differences of brain volume might be related to both hypoglycemia and hyperglycemia.

Recent reports also indicated the relation between frequent episodes of severe hypoglycemia and the increased risk of later-onset epilepsy (42, 43), and although the causative mechanisms were not elucidated, metabolic brain adaptations to frequent severe hypoglycemia might be the cause of later-onset epilepsy (44).

### Psychological Outcomes

Severe hypoglycemia is likely to provide negative psychosocial outcomes and undesirable compensatory behaviors (2, 45). The fear toward severe hypoglycemia may induce anxiety, and in many children and family members, significant degrees of anxiety possibly lead to confusion in daily living activities and inadequate management of diabetes (46). Children with type 1 diabetes and family members have risks of increased anxiety, insufficient sleep, and impaired quality of life (2, 47, 48). Fear of severe hypoglycemia, particularly during night, must be the most serious problem in family members of younger children with type 1 diabetes (49). This fear may lead them to accept high blood glucose levels, and suboptimal glycemic control with behaviors avoiding hypoglycemia resulted in inadequate glycemic control (2, 50, 51). The use of CGM and sensor-augmented pump can decrease the fear of hypoglycemia, although the studies conducted in children are limited (46, 52).

### Risk Factors for Developing Severe Hypoglycemia

Various factors can affect severe hypoglycemia. Possible risk factors for developing severe hypoglycemia are shown in Table 1.

#### Younger Age

Various studies have indicated that younger children tend to have more frequent and/or more serious episodes of hypoglycemia than later-onset patients with type 1 diabetes (53–57). Younger children are likely to have more physical activities and less consumption of food, showing fluctuating blood glucose profiles, which increase risks for developing hypoglycemia (4). They lack counterregulatory hormone responses to subsequent hypoglycemia via autonomic function (56). Deficits of counterregulatory hormone responses also cause autonomic failure. Consequently, younger children are at risk for frequent and severe hypoglycemia. Neurological damages accompanied by severe hypoglycemia are more common and more severe in younger children with type 1 diabetes (13, 14). The onset age of diabetes might play a role for early exposure to severe hypoglycemia, as earlier exposure can occur in those with younger onset of type 1 diabetes (31).

#### Nocturnal Hypoglycemia

The counterregulatory hormone responses to hypoglycemia attenuate during sleep (56, 58), and patients with type 1 diabetes tend to be less awakened by hypoglycemia compared with healthy subjects (56). The fear of nocturnal hypoglycemia often provides anxiety and emotional stress, interfering sleep and lowering quality of life in family members, which is the most common cause of distress in family members (59).

Earlier reports showed a high frequency of nocturnal hypoglycemia up to 40% during any nights in pediatric patients with type 1 diabetes (60–63), whereas recent studies have demonstrated lowering frequencies of 15–25% during any nights (63, 64). Half of these hypoglycemic events were undetected by patients themselves, families, and caregivers (60, 65). The Juvenile Diabetes Research Foundation (JDRF) found recurrent and prolonged nocturnal hypoglycemia during 8.5% of nights in both children and adults, but more extended in children (66). The use of insulin pump can decrease nocturnal hypoglycemia (67), and this is further decreased using sensor-augmented pump with the control algorithms, which can suspend basal insulin delivery with sensor-detected (68) or sensor-predicted hypoglycemia (69).

#### Impaired Awareness of Hypoglycemia

Impaired awareness of hypoglycemia is an acquired complication of insulin treatment, whereby the potency to detect the onset of hypoglycemia is decreased or absent (70). Deficit of the counterregulatory hormone responses to hypoglycemia frequently coexist. Development of impaired awareness of hypoglycemia increases the risk for severe hypoglycemia more. This condition was reported to exist in approximately a quarter of adults with type 1 diabetes. Children and adolescents had similar prevalence of 19–37% (2, 71, 72). However, a recent study has shown reduction in the prevalence over time, i.e., 33% in 2002 vs. 21% in 2015 in the same population-based cohort (73). Although the prevalence of impaired awareness of hypoglycemia has decreased, it is still a major risk factor for developing severe hypoglycemia. Patients with impaired awareness of hypoglycemia have a 6-fold increase in the prevalence of severe hypoglycemia (74).

It has been known that impaired awareness of hypoglycemia is related to decrease in glycemic thresholds for the release of counterregulatory hormones and induction of adrenergic warning signs. Korytkowski et al. (75) reported that a 2- to 3-fold decrease in the epinephrine responses was related to the loss of adrenergic warning symptoms against hypoglycemia. On the other hand, loss of autonomic symptoms precedes the neuroglycopenic symptoms, and patients are less likely to recognize hypoglycemia. Hypoglycemia tends to be prolonged when the awareness of low blood glucose is impaired. Patients can develop hypoglycemic seizures if unrecognized and prolonged conditions continue for more than 2–4 h (76).

Most events of severe hypoglycemia occur during nighttime, because sleep more strongly impairs the counterregulatory hormone responses to hypoglycemia in patients with type 1 diabetes as well as normal subjects (58). On the other hand, the glycemic threshold for neuroglycopenia does not change as much with the intensity of treatment, glycemic control, or with prior hypoglycemia (77–79).

Avoidance of severe hypoglycemia for 2–3 weeks can reverse impaired awareness of hypoglycemia (17), which is difficult to achieve in practice with current intensive insulin treatment in children with type 1 diabetes. Advanced technologies, such as the use of CGM (80) or sensor-augmented pump with control algorithms including suspend functions (68, 81), could reduce the rate of severe hypoglycemia in patients showing impaired awareness of hypoglycemia.

#### Frequent Episodes of Hypoglycemia

Most children with type 1 diabetes have isolated episodes of severe hypoglycemia; however, a few experience recurrent episodes of severe hypoglycemia. Frequent episodes of hypoglycemia are related to defective counterregulatory hormone responses to subsequent decrease in blood glucose concentrations. Therefore, prior episodes of frequent hypoglycemia are considered as an important risk factor for subsequent severe hypoglycemia (4). Both defective counterregulatory hormone responses and impaired awareness of hypoglycemia cause hypoglycemia-associated autonomic failure related to recurrent hypoglycemia, resulting in subsequent severe hypoglycemia (82–84). In DCCT, analysis of 424 intensively treated patients found that longer duration of diabetes, glycemic control, and prior severe hypoglycemia were related to the occurrence of severe hypoglycemia (85). JDRF reported that the higher rate of severe hypoglycemia was related to severe hypoglycemia that occurred in the last 6 months (86). Therefore, prior episodes of recurrent hypoglycemia can be one of the important predictors of subsequent severe hypoglycemia.

#### Glycemic Control

In the 1990s, strict glycemic control was evaluated to affect the frequency of severe hypoglycemia (18), particularly in younger children (19, 24). However, data from 2000 to 2009 in the Western Australian Children's Diabetes Database were analyzed, and there was a decline in the frequency of severe hypoglycemia, and relationship with glycemic control became weaker than previously reported (19, 21, 24). The reduction in the severe hypoglycemia may have resulted from improvement in management of diabetes during the past decades. The correlation between glycemic control and the risk of severe hypoglycemia seems to be weaker, without increased risk of severe hypoglycemia associated with improvement of glycemic control (21, 26–28). The association of a low HbA_1c_ level is not a predictable value for severe hypoglycemia in pediatric patients with type 1 diabetes (27). Therefore, adequate glycemic control can be attained without increasing episodes of severe hypoglycemia.

#### Coexisting Morbidities

Coexisting morbidities, including hypothyroidism (87), celiac disease (88), and Addison disease (89, 90), have been reported to be possible risk factors for severe hypoglycemia. The use of a gluten-free diet and adequate treatment of Addison disease and hypothyroidism can decrease the rate of severe hypoglycemia. Rarely, intentional self-administration of insulin to cause hypoglycemia, i.e., factitious hypoglycemia, can introduce recurrent and serious hypoglycemia and should be diagnosed as having psychological problems including eating disorders or psychiatric disease (91).

#### Urgent Treatment of Severe Hypoglycemia

Urgent treatment is required when severe hypoglycemia occurs and can be effectively reversed by injection of glucagon, which can be administered intravenously, intramuscularly, or subcutaneously (92, 93). Family members and caregivers have difficulties in preparation and administration of glucagon, because glucagon reconstitution with sterile water is required in the current preparations. To resolve these problems, an intranasal glucagon preparation has been tried in children (94) and adults (95) with type 1 diabetes and was revealed to be a promising alternative to intramuscular glucagon. Glucagon cannot be available in areas with limited resources, and in the areas where glucagon may not be available, glucose gel or in powder form is used.

On the other hand, glucose must be administered intravenously more than a few minutes to reverse hypoglycemia. Rapid infusion or excessive concentration (i.e., 50%) can cause an excessive osmotic alteration, leading to hyperosmolar injury of brain (96).

### Advanced Technologies for Prevention of the Occurrence of Severe Hypoglycemia and Decrease in Risk Factors for Developing Severe Hypoglycemia

Prevention of severe hypoglycemia remains one of the most critical issues in the management of pediatric patients with type 1 diabetes. Closed-loop system is probably the best technology for prevention of hypoglycemia; however, in the initial step toward closed-loop system, CGM or integrated CGM and insulin pump have enabled patients with type 1 diabetes to further decrease hypoglycemia (4). Possible technologies to prevent the occurrence of severe hypoglycemia and to decrease in the risk factors for developing severe hypoglycemia are shown in Table 1.

#### CGM

Several studies have reported that CGM can reduce hypoglycemic events with a concomitant improvement in HbA_1c_ in patients with type 1 diabetes, regardless of age (97–99). A randomized controlled multicenter study demonstrated reduction in time spent in hypoglycemia concomitant with a decrease in HbA_1c_ in both children and adults with type 1 diabetes (98). A multicenter analysis of 3,553 subjects from the German-Austrian-Swiss-Luxembourgian Diabetes Prospective Follow-up registry demonstrated that initiation and regular use of CGM in children and adolescents with type 1 diabetes were associated with reduction in both diabetic ketoacidosis and severe hypoglycemia with modest improvement in glycemic control (100). Although the use of CGM can decrease the episodes of severe hypoglycemia in adult patients (80, 101), this effect is not elucidated in pediatric patients (102). Moreover, JDRF (66) reported frequent and often prolonged hypoglycemia, particularly during nighttime, in pediatric patients with type 1 diabetes, although using CGM; i.e., hypoglycemic events occurred in 8.5% during nights, and the duration of hypoglycemia over 2 h was 23% of the nights. Adolescents have a high acoustic arousal threshold from sleep (103) and therefore could have severe hypoglycemic events during nighttime (76). Buckingham et al. (104) reported that 71% of youth wearing CGM did not respond to nighttime alarms. On the other hand, Ly et al. (105) reported that CGM with preset alarms improved epinephrine response in adolescents with type 1 diabetes, who had impaired awareness of hypoglycemia and a risk for nocturnal hypoglycemia. This study suggests that CGM might be a useful tool to relieve impaired awareness of hypoglycemia and potently avoid severe hypoglycemia in adolescents with type 1 diabetes.

Intermittently scanned CGM (isCGM; FreeStyle Libre; Abbott Diabetes Care, Alameda, CA, USA) has similar methodology to show continuous glucose measurements as ambulatory glucose profiles retrospectively at the time of checking. Glucose trend can be observed after intermittently scanning the sensor. IsCGM is approved in a number of countries for use, but there were a few clinical studies showing the effect on glycemic control in pediatric patients (106–109). These studies on isCGM have demonstrated a similar effect on maintaining adequate glycemic control as when using CGM, but decrease in the time spent in hypoglycemia seems difficult on multiple daily injections of insulin without using the advanced technologies, such as a sensor-augmented pump with low-glucose suspension or a hybrid closed-loop system (109).

#### Sensor-Augmented Pump Therapy With Low Glucose Suspension and That With Predictive Low Glucose Suspension

Sensor-augmented pump with low-glucose suspension further decreases the time spent in hypoglycemia and the occurrence of severe hypoglycemia. If the users ignore the alarm sounds, a low-glucose suspend system automatically suspends basal insulin delivery for up to 2 h in response to sensor-detected hypoglycemic events, after which basal insulin delivery is resumed at the programmed rate. A low-glucose suspend system can reduce the time of hypoglycemia, particularly during nighttime (81, 110, 111). This function also decreases moderate to severe hypoglycemic events, particularly in patients with impaired awareness of hypoglycemia (81). Furthermore, hyperglycemia, deterioration in overall glycemic control, and development to ketoacidosis are low frequencies (97, 111).

**Figure 2:**
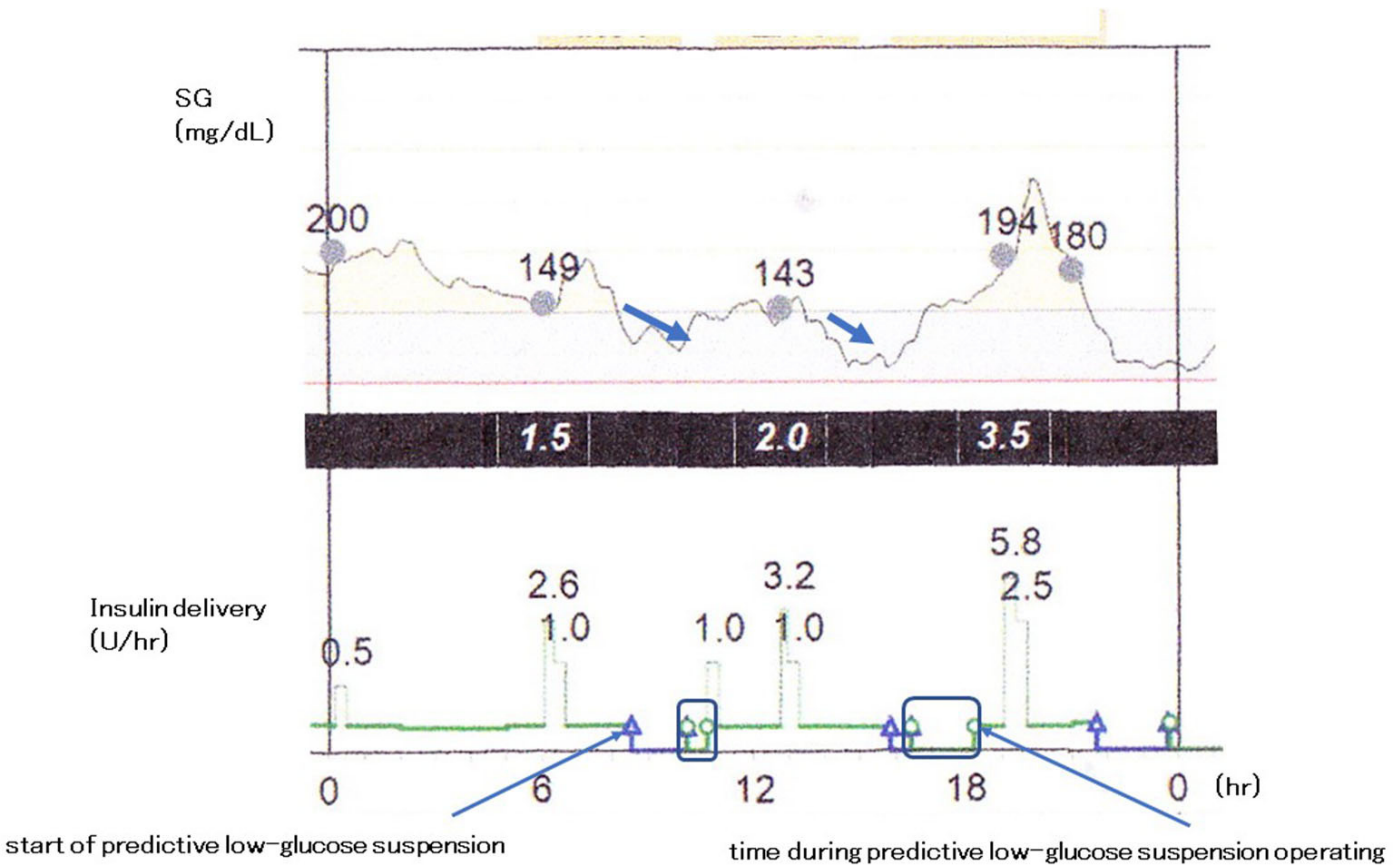
Predictive low-glucose management system. The MiniMed 640G System (Medtronic, Northridge, CA, USA).

Predictive low-glucose management system, the MiniMed 640G System (Medtronic, Northridge, CA, USA), suspends basal insulin delivery with the hypoglycemia prediction algorithm. Basal insulin delivery is usually suspended when sensor glucose level is within 3.9 mmol/L (70 mg/dL) above the patient-set low limit and is predicted to be 1.1 mmol/L (20 mg/dL) above this low limit for 30 min. When patients do not interfere, accompanied by the pump suspension, the insulin delivery resumes after the suspend period of 2 h or less at the programmed rate (Figure 2). The use of predictive low-glucose suspension more effectively decreases the rate of hypoglycemia and the risk of severe hypoglycemia in patients with type 1 diabetes (68, 68, 112–117). Buckingham et al. (117) reported that predictive low-glucose suspension prevented hypoglycemia on 75% of nights and in 84% of predicted events in adolescents and young adults with type 1 diabetes. In the study, there was mild ketosis in a few cases when the insulin pump was suspended for 1.5–2 h, and serum ketone bodies returned to normal range with resumption of basal insulin delivery. On the other hand, Abraham et al. (68) demonstrated that predictive low-glucose suspension was related to reduction in hypoglycemia than single use of sensor-augmented pump in a 6-month, multicenter, randomized controlled trial for pediatric patients with type 1 diabetes. This decline was observed both during daytime and nighttime. Episodes of hypoglycemia with a sensor-glucose value <3.5 mmol/L (63 mg/dL) for over 20 min also reduced with predictive low-glucose suspension than single use of sensor-augmented pump. Deterioration of glycemic control was not found in the use of the predictive low-glucose suspension. These findings suggest that a sensor-augmented pump therapy with low-glucose management, especially with predictive low-glucose suspension, is an important promising tool to decrease the frequency in the occurrence of hypoglycemia and the risks for severe hypoglycemia without worsening overall glycemic control in pediatric patients with type 1 diabetes.

#### Hybrid Closed-Loop System

**Figure 3:**
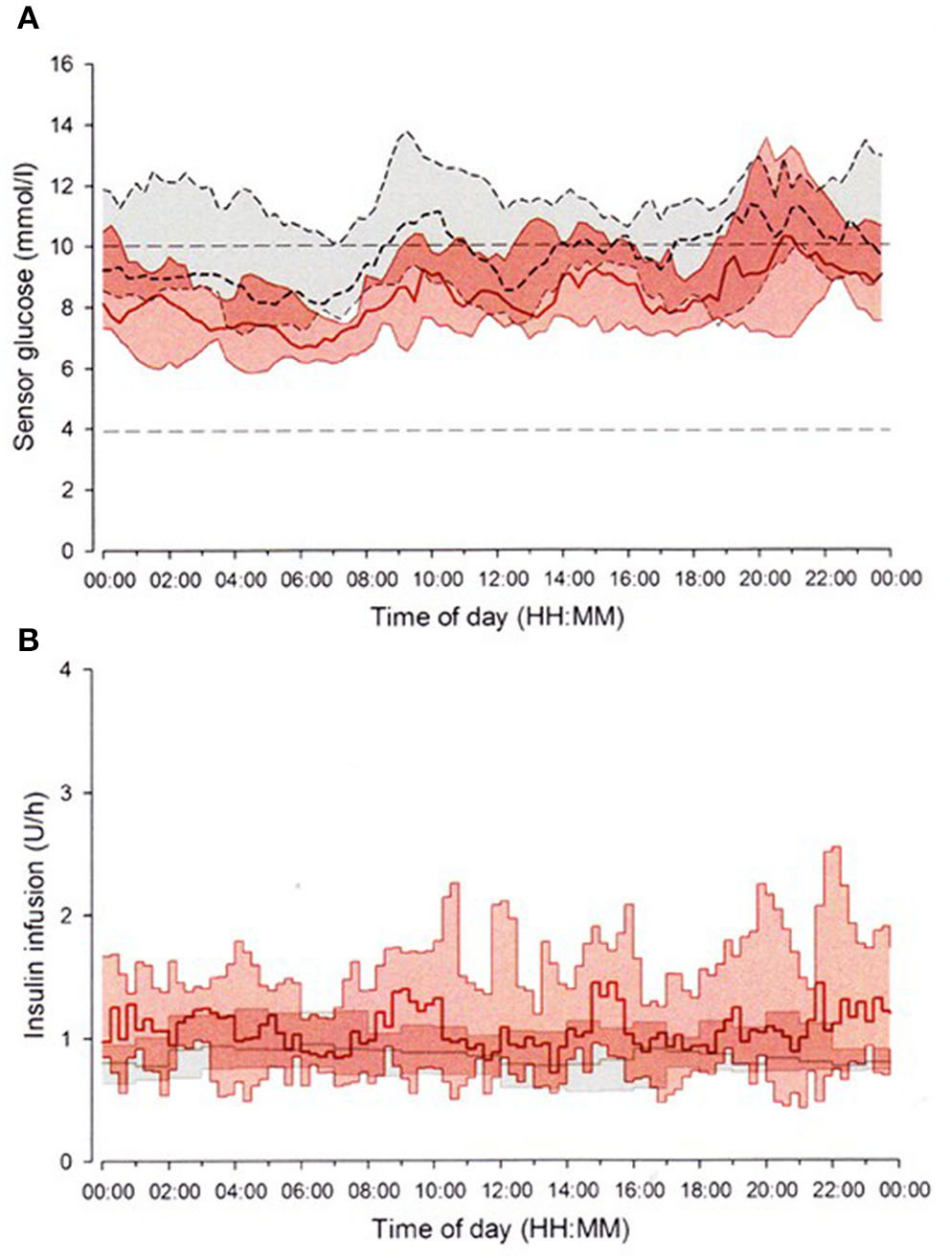
Closed-loop system. The FlorenceD2A closed-loop system (University of Cambridge, Cambridge, UK) (125). Median sensor glucose **(A)** and insulin delivery **(B)** during closed-loop insulin delivery period (solid red line and red-shaped area) and sensor-augmented pump period (dashed black line and gray-shaped area) from midnight to midnight. The glucose range 3.9–10.0 mmol/L (70–180 mg/dL) is denoted by horizontal dashed lines **(A)**.

In 2016, the Food and Drug Administration approved the first closed-loop system, the MiniMed 670G System (Medtronic), for patients 14 years or older in the United States. Hybrid closed-loop system, commonly referred to as an artificial pancreas, is an automated insulin delivery management, combined with CGM and insulin delivery without patient intervention. The system uses a proprietary proportional-integral-derivative controller with insulin feedback to calculate insulin dosages continually according to CGM levels (118–120). Several studies have been handled on the closed-loop systems and demonstrate improvement of glycemic control with decrease in the rate of hypoglycemia and the occurrence of severe hypoglycemia in both adults and children, especially at nighttime (121–125). In an open-label, randomized, crossover study design, the use of a closed-loop system significantly increased the time spent in the target range [3.9–10 mmol/L (70–180 mg/dL)], whereas the time spent in hypoglycemic range significantly decreased [<3.9 mmol/L (<70 mg/dL)] during both daytime and nighttime in adolescents with type 1 diabetes (Figure 3) (125). The use of hybrid closed-loop system is in general effective and safe particularly at nighttime, and allows enough sleep and reduces the burden of diabetes management during overnight. The closed-loop system must be one of the most promising technologies to attain optimal glycemic control with minimizing the episodes of hypoglycemia, as well as occurrence of severe hypoglycemia. Although the majority of the systems include single-hormone insulin, dual-hormone systems, which infuse both insulin and glucagon, have also been in the research phase (126, 127).

In summary, the incidence of severe hypoglycemia has been markedly declined in recent years, but still a lethal condition. Minimizing the risk factors for development of severe hypoglycemia is an important objective to prevent the occurrence of severe hypoglycemia in pediatric patients with type 1 diabetes. The new concept of time spent within target glucose range (time in range) will be in general used to evaluate the glucose trend and the quality of metabolic control (128, 129). Achieving the target range [3.9–10 mmol/L (70–180 mg/dL)] more than 70% with minimizing severe hypoglycemia <1% is crucial in the management of not only adults but also children and adolescents with type 1 diabetes (128). This can be achieved through advanced diabetes technologies even in pediatric patients.

## Author Contributions

The author confirms being the sole contributor of this work and has approved it for publication.

## Conflict of Interest

The author declares that the research was conducted in the absence of any commercial or financial relationships that could be construed as a potential conflict of interest.
